# 

**Published:** 1872-07

**Authors:** 


### ARTICLE IV.

### *American Dental Association.*

The American Dental Association will commence its Twelfth Annual Session at Niagara Falls, Tuesday, the 6th day of August, at 10 A. M. The officers of the Association constitute the committee for securing reduced rates of fare. They are:

G. H. Cushing, Chicago; C. E. Francis, New York; J. R. Walker, New Orleans; J. A. Salmon, Boston; M. S. Dean,

Chicago; W. H. Goddard, Louisville; A. B. Robbins, Meadville, Pa.; G. B. McDonneld, Conneantville, Pa.; S. B. Palmer, Syracuse, New York; W. W. Allport, Chicago; E. A. Bogue, New York; Isaiah Forbes, St. Louis; J. S. Knapp, New Orleans; J. Taft, Cincinnati.

The following were appointed as a Special Committee to prepare a circular on Popular Education: S. B. Palmer, Homer Judd and M. S. Dean.

Drs. Homer Judd, J. Taft, W. H. Atkinson, L. D. Shepard and Wm. Dutch, were appointed a Special Committee on Local Societies and for conference with the profession. The Standing Committees are as follows:

*Dental Physiology.*—J. H. McQuillen, M. S. Dean and C. F. Wheeler.

*Dental Pathology and Surgery.*— Homer Judd, W. H. Atkinson and C. Stoddard Smith.

*Dental Histology and Microscopy.*—R. W. Varney, J. Taft and W. W. Allport.

*Dental Chemistry.*—D. S. Dickerman, H. A. Smith and J. J. Birge.

*Dental Therapeutics.*—H. L. Sage, J. R. Walker and W. N. Morrison.

*Operative Dentistry.*—C. E. Francis, E. A. Bogue and William Dutch.

*Mechanical Dentistry.*—J. A. Salmon, F. Hickman and J. H. Smith.

*Dental Education.*—A. B. Robbins, C. C. Chittenden and J. N. Crouse.

*Prize Essays.*—Geo. A. Mills, E. Floyd and R. Huey.

*Dental Ætiology.*—W. N. Bronson, L. D. Shepard and G. R. Thomas.

A letter has just been received from Dr. G. B. McDonneld, of the Committee of Arrangements, stating that he had made arrangements at the International Hotel, Cataract House and Spencer House for a reduction in the rates of board of Dentists fifty cents per day, also a reduction in several other items of expense at that place. No arrangements have yet been made with the agents of railroads to secure reduced rates of fare, but it is expected that each one of the Committee, whose names are given above, will give their attention to this matter very soon.

The transactions for the year 1871 will not be published before the next coming meeting of the Association.

M. S. Dean, Secretary.

### EDITORIAL DEPARTMEMT.

*Southern Dental Association.*—The fourth annual meeting of this Association will be held in Richmond, Va., commencing on Tuesday, July 30th, 1872, at 10 o'clock A.M. We especially urge Southern practitioners to be present at this meeting, as it will be held at a point accessible to all residing North or South of it, by rail or water. From Baltimore and points North and East, the passage can be made by rail or water, a magnificent line of steamers running daily, and the route, one unsurpassed for scenery and historical events. The following Standing Committees were appointed at the last annual meeting for the present year:

*Executive Committee.*—Drs. H. M. Grant, Va.; Jas. F. Thompson, Va.; S. G. Holland, Ga.; E. A. Herman, Tenn., and T. T. Moore, S. C.

*Committee on Membership.*—Drs. Jas. F. Thompson, Va.; J. B. Patrick, S. C., and H. C. Jones, Va.

*Committee on Publication.*—Drs. Sam'l A. White, Ga.; E. M. Allen, Ga., and F. J. S. Gorgas, Md.

*Committee on Dental Education.*—Drs. J. P. H. Brown, Ga; Geo. H. Winkler, S. C., and Jno. G. Angell, La.

*Committee on Physiology and Surgery.*—Drs. W. T. Arrington, Tenn.; W. H. Atkinson, N. Y., and Jas. S. Knapp, La.

*Committee on Histology and Microscopy.*—Drs. S. P. Cutler, Miss; G. T. Barker, Pa., and Wm. Reynolds, S. C.

*Committee on Dental Chemistry.*—Drs. B. F. Arrington, N. C.; W. H. Morgan, Tenn., and J. G. McAuley, Ala.

*Committee on Dental Therapeutics.*—Drs. N. W. Kingsley, N. Y.; W. H. Burr, Ga., and Sam'l Rambo, Ala.

*Committee on Operative Dentistry.*—Drs. E. A. Herman, Tenn.; A. C. Ford, Ga., and W. H. Shadoan, Ky.

*Committee on Mechanical Dentistry.*—Drs. B. A. Rodrigues, S. C.; H. J. Royal, Ga., and G. F. S. Wright, S, C.

*Committee on Dental Literature.*—Drs. F. J. S. Gorgas, Md.; W. C. Wardlaw, S. C., and E. Floyd, N. C.

*Committee on Voluntary Essays.*—Drs. H. A. Lowrance, Ga.; Sam'l A. White, Ga., and S. J. Cobb, Tenn.

---

*A New Appointment.*—Dr. James B. Hodgkin, of Alexandria, Va., a graduate of the class of 1869, has been appointed Demonstrator of Mechanical Dentistry in the Baltimore College of Dental Surgery.

Dr. Hodgkin has been in successful practice in Alexandria, and is in every respect well qualified for the duties of his new position.

*Virgina State Dental Association.*—An adjourned meeting of the "Virgina State Dental Association," will be held at the rooms of Dr. Wood, in the city of Richmond, July 30th, 1872.

Arrangements have been made with most of the Rail Roads and Hotels, for reduced fare. All attending will have the advantage of being present, at the sessions of the Southern Dental Association, which meets there at the same time. We urge attendance.

